# Unmasking the Long-Term Effects of COVID-19 With a Focus on Chronic Fatigue Syndrome: A Community-Based Study From India

**DOI:** 10.7759/cureus.80597

**Published:** 2025-03-14

**Authors:** Arti Gupta, Vishnu Rajan, Rajeev Aravindakshan, Pulla Sirisha

**Affiliations:** 1 Department of Community and Family Medicine, All India Institute of Medical Sciences Mangalagiri, Mangalagiri, IND

**Keywords:** chronic fatigue syndrome (cfs), community survey, covid 19, covid scenario, long covid, post-covid-19 syndrome

## Abstract

Background

The COVID-19 pandemic has highlighted the enduring health burden of long COVID, scientifically termed the postacute sequelae of severe acute respiratory syndrome coronavirus 2 (SARS-CoV-2) infection or PASC. Long COVID manifests as diverse symptoms affecting multiple organ systems, significantly impacting individuals globally, and especially underrepresented rural populations.

Objective

This study aimed to evaluate the prevalence of chronic fatigue syndrome (CFS) among laboratory-confirmed COVID-19 patients in rural India, focusing on identifying its predictors and the implications for healthcare systems in resource-limited settings.

Methodology

Retrospective data analysis was conducted on the follow-up visits of post-COVID-19 patients in the field practice area of AIIMS Mangalagiri, Nuthakki, Andhra Pradesh, India. The study involved 500 COVID-19 survivors diagnosed via reverse transcription polymerase chain reaction (RT-PCR) between 2023 and 2024. Data was collected via a semi-structured questionnaire to retrieve socio-demographic and clinical parameters, while CFS severity was assessed using a validated scoring system. Multivariable logistic regression and path analysis were used to examine the associations between the predictors of CFS.

Results

The prevalence of CFS was seen in 107 patients (21.4%; 95% CI: 18.01-25.22). Risk factors included older age (adjusted odds ratio or aOR: 15.90 for ≥60 years), female gender (aOR: 1.90), and comorbidities (aOR: 3.92). Common symptoms observed were fatigue in 180 (36%), joint pain in 185 (37%), and muscle pain in 182 (36.4%) patients. There was no significant association between vaccination and CFS.

Conclusion

The study underscores the substantial burden of post-COVID fatigue in rural populations, with female patients, older adults, and those with comorbidities being at greater risk. Tailored healthcare interventions and proactive post-COVID monitoring are critical to address this challenge. Future research should explore the underlying mechanisms and assess the role of vaccination in mitigating the post-COVID sequelae.

## Introduction

The emergence of severe acute respiratory syndrome coronavirus 2 (SARS-CoV-2) triggered a global health crisis, presenting unprecedented challenges in understanding infectious diseases and their long-term consequences. While the initial focus of the pandemic was on addressing immediate concerns through vaccination efforts and strict public health measures, another pressing issue surfaced - the enduring effects of COVID-19, widely known as "Long COVID." Long COVID has become a major global health concern, and is scientifically termed as postacute sequelae of SARS-CoV-2 Infection (PASC) [1]. Studies indicate that at least 10% of the individuals who contract COVID-19 experience prolonged and sometimes debilitating symptoms, encompassing over 200 distinct manifestations affecting multiple organ systems [2]. This prolonged impact knows no boundaries and affects the lives of over 65 million individuals worldwide. The World Health Organization defines long COVID as starting three months after the initial COVID-19 infection [3]*,* in which commonly reported symptoms are fatigue, memory problems, shortness of breath, and sleep disorders [4]. There is a significant overlap with chronic fatigue syndrome (CFS), which is one of the defining features of long COVID [3]. The prevalence of long COVID-19 differs across patient groups, ranging from 10-30% in non-hospitalized cases to an alarming 50-70% in hospitalized cases, with 10-12% of vaccinated individuals also being affected [5]. These persistent effects span a wide range of adverse outcomes, including cardiovascular complications, thrombotic and cerebrovascular disorders, type 2 diabetes, myalgic encephalomyelitis/chronic fatigue syndrome (ME/CFS), and dysautonomia, particularly postural orthostatic tachycardia syndrome (POTS) [3].

Numerous hypotheses have been proposed to understand the origins of long COVID, including the persistence of SARS-CoV-2 in tissues and immune dysregulation, occasionally with the reactivation of other pathogens such as Epstein-Barr virus and human herpesvirus-6 [6]. Understanding the pathogenesis of long COVID is crucial for developing effective treatment strategies and interventions. Recent studies have highlighted immune system irregularities associated with long COVID, including elevated levels of non-classical monocytes, activated B cells, double-negative B cells, and IL-4 and IL-6-secreting CD4+ T cells. Additionally, reductions in conventional dendritic cells, exhausted T cells, and cortisol levels have been observed in affected individuals [7].

India's diverse socio-demographic profile presents unique challenges, including significant healthcare accessibility issues, rural-urban disparities, and a substantial burden on primary healthcare infrastructure. Chippa et al. (2022) reported that long COVID has been observed in at least 10% of the individuals recovering from SARS-CoV-2 infections, with the prevalence potentially reaching 50% to 70% among hospitalized cases [8]. Similarly, a multicentric study identified older age, female gender, and pre-existing comorbidities as significant predictors of prolonged symptoms [9]. Another study conducted in rural Indian settings emphasized the disparity in symptom management and access to healthcare resources between urban and rural populations, further complicating the burden of post-COVID-19 sequelae [10]. Vaccination has been hypothesized to reduce the severity of post-COVID-19 outcomes; however, data from Indian populations remain scarce [11]. Our study aims to explore this association. Comprehensive studies focusing on the post-COVID syndrome in India are essential to understanding its burden, risk factors, and implications for public health policies tailored to the Indian context.

The primary objective of this study is to determine the prevalence of CFS and identify risk factors contributing to its development among laboratory-confirmed COVID-19 patients attending specialized post-COVID-19 clinics and outreach care programs in a rural healthcare setting in India. Our study provides a detailed examination of the interactions between SARS-CoV-2, long COVID, and CFS, aiming to identify risk factors associated with CFS development and exploring potential management strategies in resource-limited settings.

## Materials and methods

Study design, setting, and procedure

This retrospective study was conducted at the Centre for Rural Health AIIMS Mangalagiri (CRHA) Nuthakki, Guntur District, Andhra Pradesh, India, from July 2023 to January 2024. The study included COVID-19 survivors diagnosed with the infection using a reverse transcription polymerase chain reaction (RT-PCR) test. Routine follow-up visits with post-COVID-19 patients in the field practice area of CRHA were carried out, and they were evaluated for new-onset symptoms, signs, and the presence of CFS, and their severity was assessed using a CFS severity score. The vaccination status was retrieved from their medical records. Follow-up visits were scheduled monthly or as needed, depending on when patients reported to the clinic's outreach area. Strict privacy standards were adhered to during data collection, and the information obtained from the CRHA Health and Demographic Surveillance System (HDSS) lacked personal identifiers. An Institutional Ethics Committee (IEC) approval for waiver of consent was obtained to authorize the study, data analysis, and subsequent reporting (AIIMS/MG/IEC/2023-24/01) in accordance with ethical guidelines for retrospective studies.

Study tools

A semi-structured questionnaire was used to collect data on socio-demographic characteristics, comorbidities, history of the COVID-19 illness, vaccination status, and newly-emerging symptoms. The severity of the post-COVID-19 symptoms and fatigue was evaluated using a validated CFS score [12]. Participants rated fatigue severity and eight additional criteria over the past six months using an anchored ordinal scale (0 = no symptom, 1 = trivial, 2 = mild, 3 = moderate, 4 = severe). Fatigue ratings of three or four were used to classify participants as “Fatigued,” while lower scores categorized them as “Not fatigued.”

Sample size and statistical analysis

The sample size was estimated using OpenEpi [13], assuming an anticipated incidence of 25% [14], a relative precision of 4%, and a 95% confidence interval, yielding a minimum requirement of 450 participants. Accounting for a 10% non-response rate, the final sample size was set at 500. The data collection forms were created in the Epicollect5 [15] mobile application, and the data sheets were exported to MS Excel (Microsoft Corp., Redmond, WA, US). The data analysis was carried out using STATA version 17 (StataCorp LLC, College Station, TX, US), and R version 4.4.1 (The R Foundation, Vienna, Austria). Categorical variables were summarized using frequencies and proportions, while continuous variables were described using mean with standard deviation or median with IQR based on the normality of data. A Pearson chi-squared test and an independent sample t-test Mann-Whitney were used to compare the differences in outcomes. Multivariable logistic regression was used to find the determinants and their association with post-COVID-19 fatigue. The regression model, generated through path analysis using the lavaan module in R, was employed to explain the complex relationships between predictor variables, including comorbidities, age, gender, and COVID-19 vaccination, and CFS. The multivariable regression model was adjusted for age, gender, history of comorbidities, random blood sugar and blood pressure. The indirect effect, i.e., the influence of an independent variable on CFS through the other mediator variables, was examined in the path analysis.

## Results

The study included 500 participants with a mean age of 41.62 years (SD=16.92), with the majority being males (n=265; 53%). A comparison of the socio-demographic characteristics of COVID-19-related details and clinical parameters between participants with and without fatigue is presented in Table 1. Participants with fatigue were significantly older (49.1±14.1 years) than those without it (39.5±17.1 years). A higher proportion of females (n=134; 26.8%) reported fatigue compared to males (n=83; 16.6%).

**Table 1:** Socio-demographic characteristics and details of the COVID-19 infection(s) among the study participants (n=500)

Characteristics	Categories	Normal, n (%); total n=393	Fatigue, n (%); total n=107	p-value
Mean Age (Mean ± SD)	-	39.5 ± 17.1	49.1 ± 14.1	<0.001
Age groups (in years)	<18 years	37 (97.4)	1 (2.6)	<0.001
	19-44	206 (86.6)	32 (13.4)	
	45-59	100 (68.0)	47 (32.0)	
	>60 years	50 (64.9)	27 (35.1)	
Gender	Female	172 (73.2)	63 (26.8)	0.005
	Male	221 (83.4)	44 (16.6)	
Year of COVID-19 infection	2019	2 (100.0)	0 (0.0)	0.29
	2020	11 (73.3)	4 (26.7)	
	2021	373 (79.2)	98 (20.8)	
	2022	6 (54.5)	5 (45.5)	
	2023	1 (100.0)	0 (0.0)	
Frequency of COVID-19 infection (number of times)	One time	393 (78.8)	106 (21.2)	0.055
	Two times	0 (0.0)	1 (100.0)	
History of COVID-19 infection in family members	No	131 (78.5)	36 (21.5)	0.95
	Yes	262 (78.7)	71 (21.3)	
Death of family member due to COVID-19	No	369 (78.3)	102 (21.7)	0.57
	Yes	24 (82.8)	5 (17.2)	
Vaccination against COVID-19	No	26 (89.7)	3 (10.3)	0.13
	Yes	367 (77.9)	104 (22.1)	
History of comorbidities	No	388 (79.5)	100 (20.5)	0.002
	Yes	5 (41.7)	7 (58.3)	
New symptoms after COVID-19	No	341 (87.0)	51 (13.0)	<0.001
	Yes	52 (48.1)	56 (51.9)	
Currently under treatment for symptoms	No	389 (80.0)	97 (20.0)	<0.001
	Yes	4 (28.6)	10 (71.4)	
Random blood sugar (mg/dL)	-	113.1 ± 47.8	137.1 ± 68.0	<0.001
Systolic blood pressure (mmHg)	-	118.3 ±12.6	123.4 ± 13.6	<0.001
Diastolic blood pressure (mmHg)	-	80.9 ± 37.6	81.0 ± 8.9	0.97

**Figure 1:**
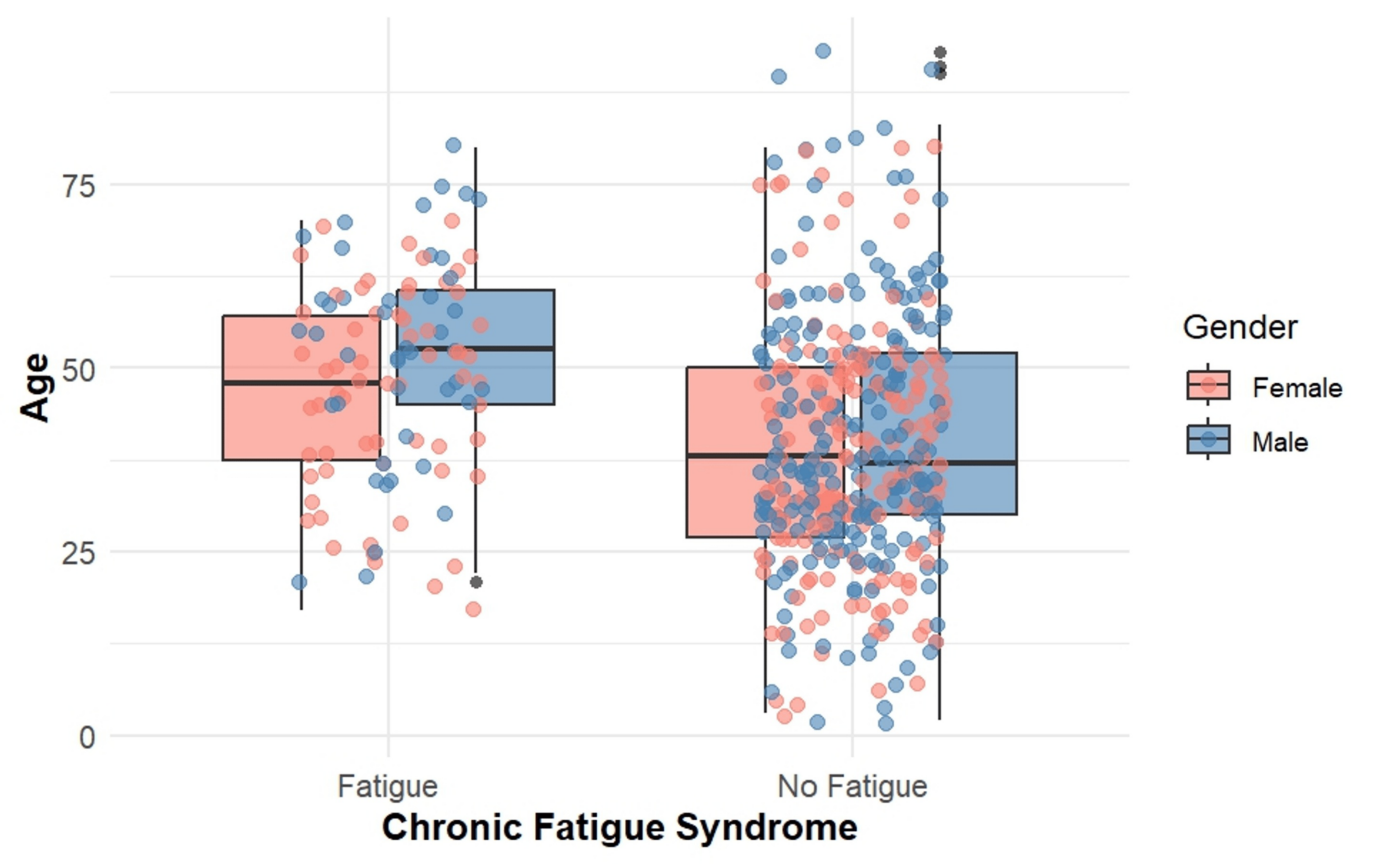
Relationship of chronic fatigue syndrome with the age and gender of the participants

The relationship between CFS and the age and gender of the participants is depicted in Figure 1.

Those who were infected more than once (n=291; 58.3%) or reported new symptoms after COVID-19 (n=259; 51.9%) were more likely to experience fatigue. Vaccinated participants showed a slightly higher fatigue prevalence (n=110; 22.1%) than unvaccinated ones (n=51; 10.3%). Higher proportions of individuals with comorbidities (n=291; 58.3%) or under treatment for post-COVID-19 symptoms (n=357; 71.4%) had fatigue. Random blood sugar and systolic blood pressure levels were elevated in the group with fatigue, while diastolic pressure differences were minimal. The prevalence of CFS was found in 107 patients (21.4%) (95% CI: 18.01 - 25.22). Joint pain was the most frequently reported symptom (n=185; 37%), followed by muscle pain (n=182; 36.4%), fatigue after mild exertion (n=180; 36%), and headache (n=160; 32%). Some of the less reported symptoms included sore throat (n=7; 1.4%), reduced memory (n=29; 5.8%) and reduced sleep (n=56; 11.2%).

Participants aged 45 to 59 years had significantly higher odds of suffering from CFS compared to those under 18 years, with an adjusted odds ratio (aOR) of 12.89 (95% CI: 1.60-103.66, p=0.01). Similarly, participants aged 60 years and above exhibited an even greater risk, with an aOR of 15.90 (95% CI: 1.88-134.19, p=0.01). Female participants were nearly twice as likely to develop CFS compared to males, with an aOR of 1.90 (95% CI: 1.20-3.03, p=0.006) (Table 2)

**Table 2:** Multivariable analysis showing factors associated with chronic fatigue syndrome

Characteristic	Fatigue, n (%)	Adjusted odds ratio (aOR)	95% CI	p-value
Age groups (in years)				
<18 years	1 (2.63)	Ref	-	-
19-44	32 (13.45)	5.08	0.64 - 0.36	0.12
45-59	47 (31.97)	12.89	1.60 - 103.66	0.01
>60 years	27 (35.06)	15.90	1.88 - 134.19	0.01
Gender				
Male	44 (16.60)	Ref	-	-
Female	63 (26.81)	1.90	1.20 - 3.03	0.006
History of any comorbid illnesses				
No	100 (20.49)	Ref	-	-
Yes	7 (58.33)	3.92	1.12 - 15.2	0.036
History of at least one or two COVID-19 vaccinations				
No	3 (10.34)	-	-	-
Yes	104 (22.08)	1.52	0.45 - 6.99	0.5
Mean blood sugar(mg/dL)	-	1.00	1.00, 1.01	0.2
Systolic blood pressure (mmHg)	-	1.01	0.99, 1.03	0.4
Diastolic blood pressure (mmHg)	-	0.99	0.95, 1.00	0.4

**Figure 2:**
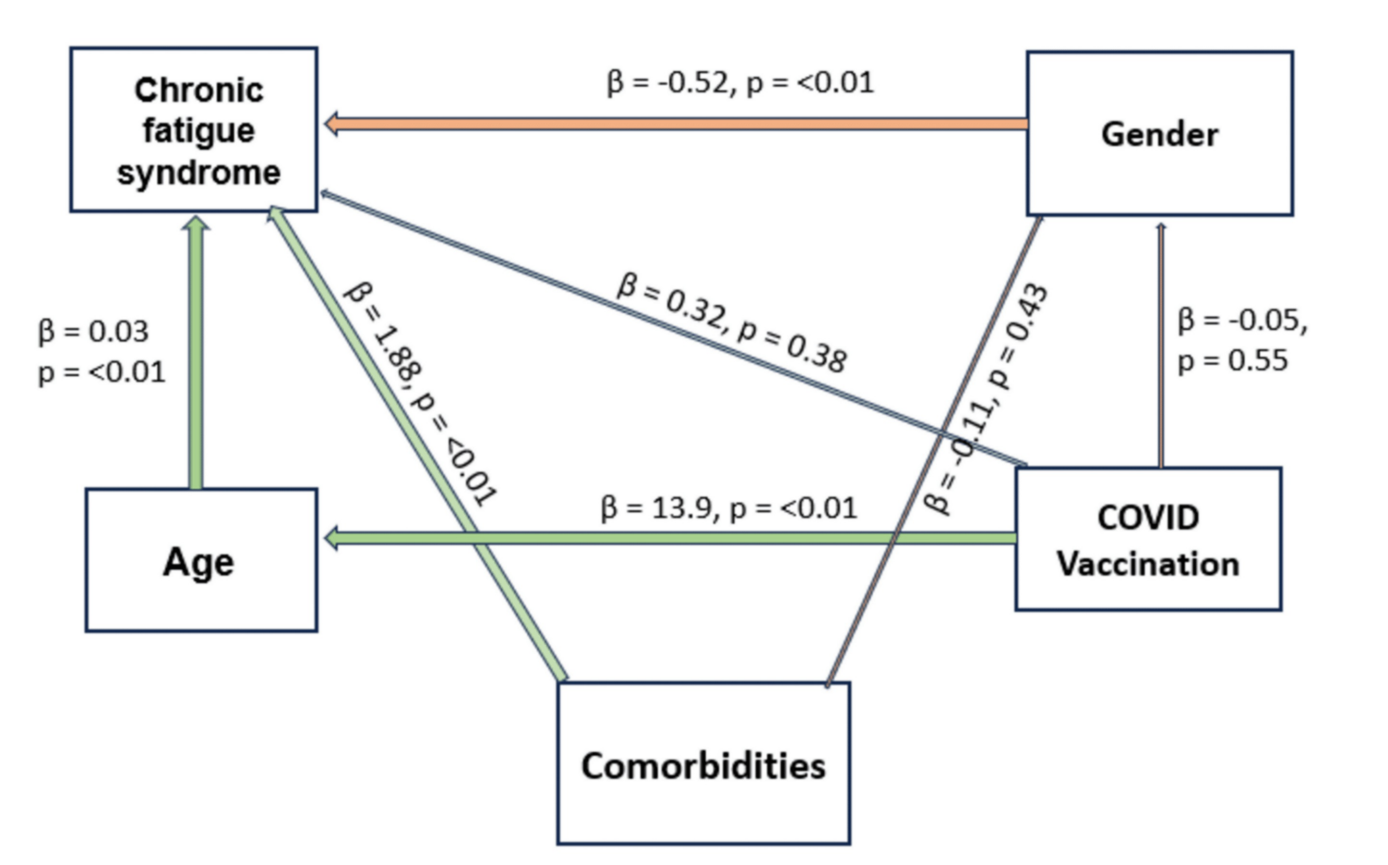
Path analysis showing the relationship between chronic fatigue syndrome and its predictors

The path analysis revealed that comorbidities were significantly associated with higher CFS scores (b =1.884, p=<0.01). Age was also positively associated with CFS scores indicating that older individuals reported a higher impact of fatigue. Gender had a significant negative association with CFS scores where male patients reported a lower impact of fatigue compared to female ones (b = -0.52, p=<0.01). In contrast, COVID-19 vaccination status did not significantly predict CFS (b=0.36, p=0.3) (Figure 2).

## Discussion

The study aimed to determine the prevalence of post-COVID-19 syndrome and identify the predictors of CFS among COVID-19 survivors in a rural healthcare setting. Our findings reveal that 21.4% of the participants experienced post-COVID-19 fatigue, with females, older adults, and those with comorbidities at significantly higher risk. This prevalence of post-COVID-19 fatigue is lower compared to other studies, which reported a prevalence ranging from 40% to 80% [14,16]. This difference may be attributed to the community-based approach utilized in our study. In a meta-analysis, fatigue and headache were found to be the most common symptoms during the post-COVID-19 period [17]. This variability in prevalence is likely influenced by differences in sample characteristics, definitions of fatigue, and assessment methods. Our study utilized a validated CFS score, ensuring reliable identification of cases based on symptom severity and persistence over six months. Even though the prevalence in our study is lower, it is consistent with studies focused on less severe disease and rural areas [10,17].

The study identified significant associations between age, gender, and fatigue. Participants aged 45-59 years (aOR: 12.89) and those aged ≥60 years (aOR: 15.90) were at markedly higher risk compared to younger individuals, similar to findings from previous research that linked older age to prolonged post-viral syndromes [18]. The physiological changes and immune dysregulation associated with ageing may contribute to this increased vulnerability in older age groups [19]. Aligning with our study's findings, several studies have reported that women tend to be at greater risk for long-term COVID-19 manifestations irrespective of the baseline severity of the disease [20,21]. Prior studies suggest that women may be more prone to long COVID syndromes due to hormonal influences that sustain hyperinflammatory states even after acute illness. Moreover, female patients have been reported to produce more IgG antibodies during the early phase of the disease [22]. A path analysis revealed that comorbidities and age were the strongest predictors of CFS, while male patients reported significantly lower fatigue scores than females. Vaccination status showed no significant association with CFS scores or other variables, although vaccinated individuals were generally older. The direct effect of gender on fatigue remained significant, reinforcing its role as a primary determinant.

Comorbidities significantly increased the likelihood of fatigue syndrome, with an aOR of 3.92. This aligns with research demonstrating that pre-existing conditions such as diabetes, hypertension, and cardiovascular disease exacerbate post-COVID-19 sequelae. Participants reporting new symptoms after COVID-19, such as joint pain, muscle pain, and fatigue after mild exertion, also showed higher odds of developing chronic fatigue syndrome [23]. Comorbidities can mediate the relationship between age and fatigue severity, as older individuals often have pre-existing conditions that exacerbate fatigue symptoms post-COVID-19 [24]. Gender differences also play a role, with studies indicating that women may experience more severe fatigue due to hormonal influences and immune response variations [25]. These factors contribute to the variance in fatigue severity, highlighting the need for gender-specific and age-adjusted approaches in managing post-COVID-19 fatigue. 

We could not find any significant association between vaccination and post-COVID-19 syndrome. This could be partly explained by the broad definition of CFS and insufficient follow-up time. Previous studies have presented mixed results on the role of vaccination in mitigating long-term COVID-19 symptoms. While vaccination reduces the severity of acute illness and hospitalization, its effect on the postacute sequelae remains less clear [26,27]. Vaccination, particularly with mRNA-based vaccines, has been shown to attenuate systemic inflammation and limit viral replication, potentially reducing the risk of persistent symptoms [28].

The apparent lack of association of vaccination on post-COVID-19 fatigue severity might be attributed to several factors. Firstly, studies have shown mixed results regarding the vaccine's impact on the symptoms of long COVID, including fatigue. Some research indicates that vaccination post-infection may reduce symptom severity, while other studies have observed minimal impact [29]. Potential reasons for these differences include differences in sample sizes and the specific timing of vaccination relative to infection, which could affect immune response and symptom resolution [30]. Additionally, the effectiveness of vaccines may vary across different populations, influenced by age, underlying health conditions, and virus variants [31]. These variables underscore the need for more targeted research to identify which subgroups benefit the most from vaccination in mitigating symptoms of long COVID-like fatigue.

This study demonstrates several notable strengths. A robust sample size provided adequate statistical power to uncover associations and trends related to post-COVID-19 fatigue. The use of validated tools, such as the CFS severity score, ensured a standardized and reliable assessment of symptoms and fatigue levels. Furthermore, the analysis incorporated advanced statistical techniques, including multivariable logistic regression and pathway analysis, which allowed for a comprehensive understanding of the factors influencing post-COVID-19 fatigue and the interplay between variables such as gender, age, comorbidities, and vaccination status.

However, the study is not without limitations. The retrospective design limits causal inference and inherently introduces the possibility of recall bias, as participants may not accurately remember or report details about their symptoms, illness severity, or medical history. While the inclusion of participants from a rural setting provides valuable insights into a population that may often be underrepresented in research, it also limits the generalizability of the findings. Urban populations, who may have different exposures, healthcare access, and health-seeking behaviors, might exhibit different patterns of post-COVID-19 outcomes.

## Conclusions

This study provides valuable insights into the burden and predictors of post-COVID-19 fatigue in a rural Indian population. Healthcare systems in rural areas should prioritize long-term monitoring of COVID-19 survivors, particularly older adults and individuals with comorbidities, to detect and manage post-COVID-19 fatigue. Its high prevalence highlights the need for targeted interventions, particularly for high-risk groups such as older adults, females, and individuals with comorbidities. Integrating post-COVID-19 care into existing healthcare services, especially in rural settings, can help address these long-term sequelae. Although the prevalence of long COVID is high, these results should be interpreted with caution, as self-reported symptoms and healthcare-seeking behavior may cause potential confounding effects. Further research is needed to explore the biological mechanisms underlying fatigue and to assess the long-term benefits of vaccination on post-COVID-19 outcomes. To better understand the mechanisms underlying post-COVID-19 fatigue, further longitudinal cohort studies are needed to identify potential risk factors. Additionally, biomarker analysis can be valuable in pinpointing physiological changes linked with its persistence. 

## Figures and Tables

**Figure 3:**
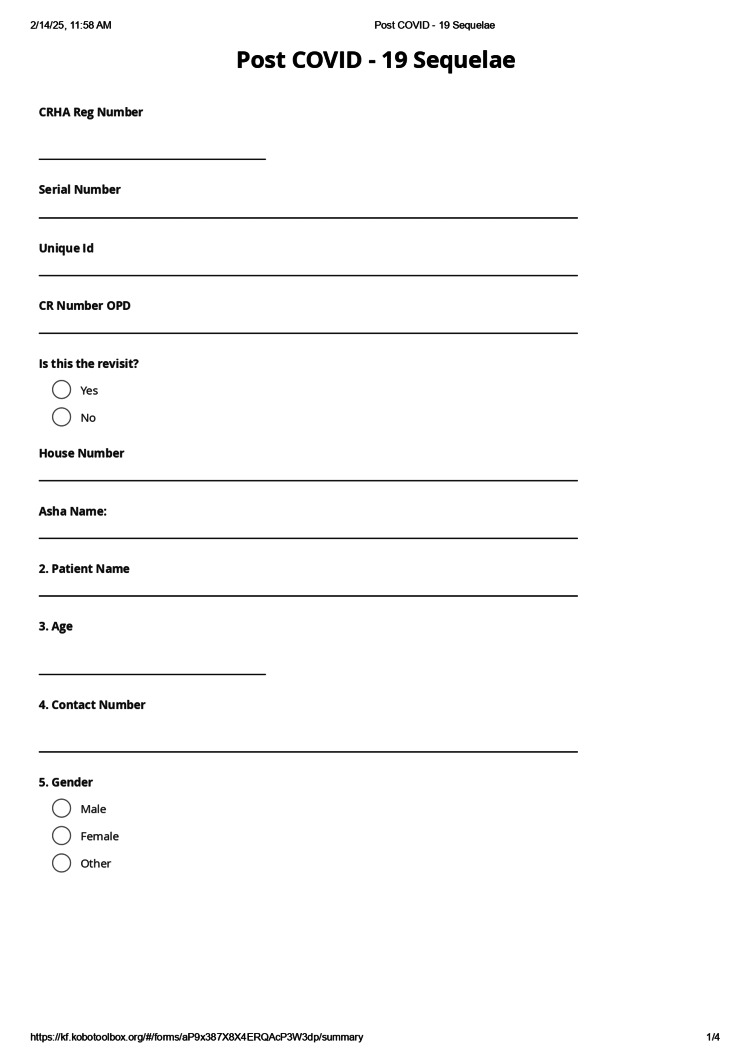
Post-COVID-19 questionnaire (page 1)

**Figure 4:**
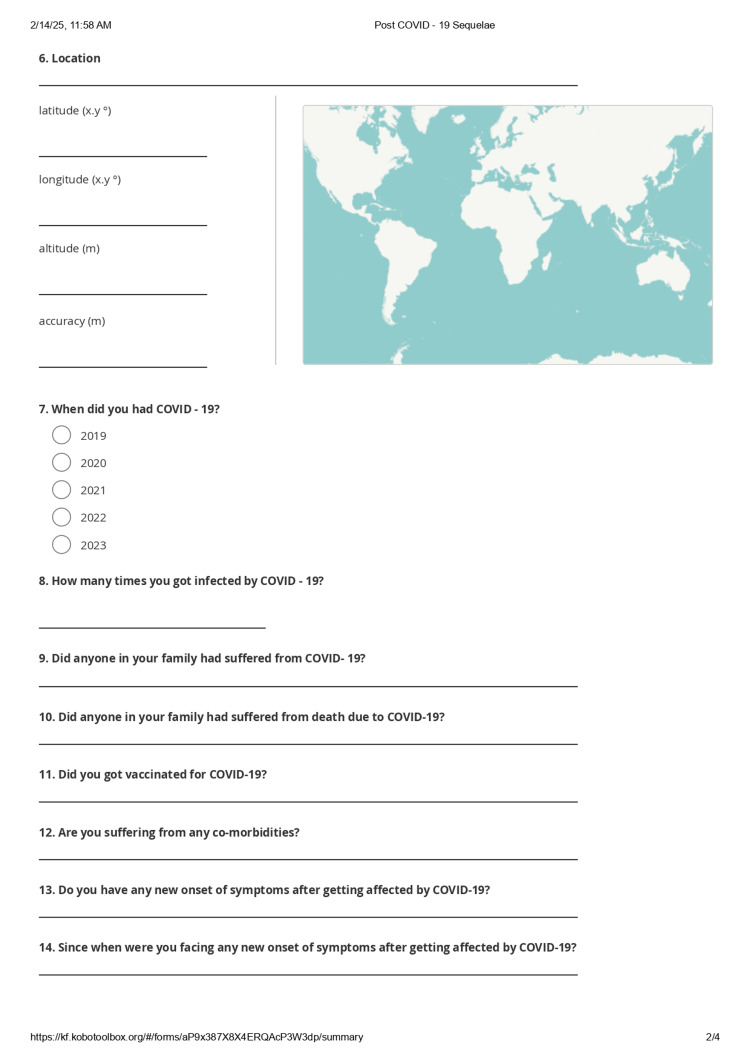
Post-COVID-19 questionnaire (page 2)

**Figure 5:**
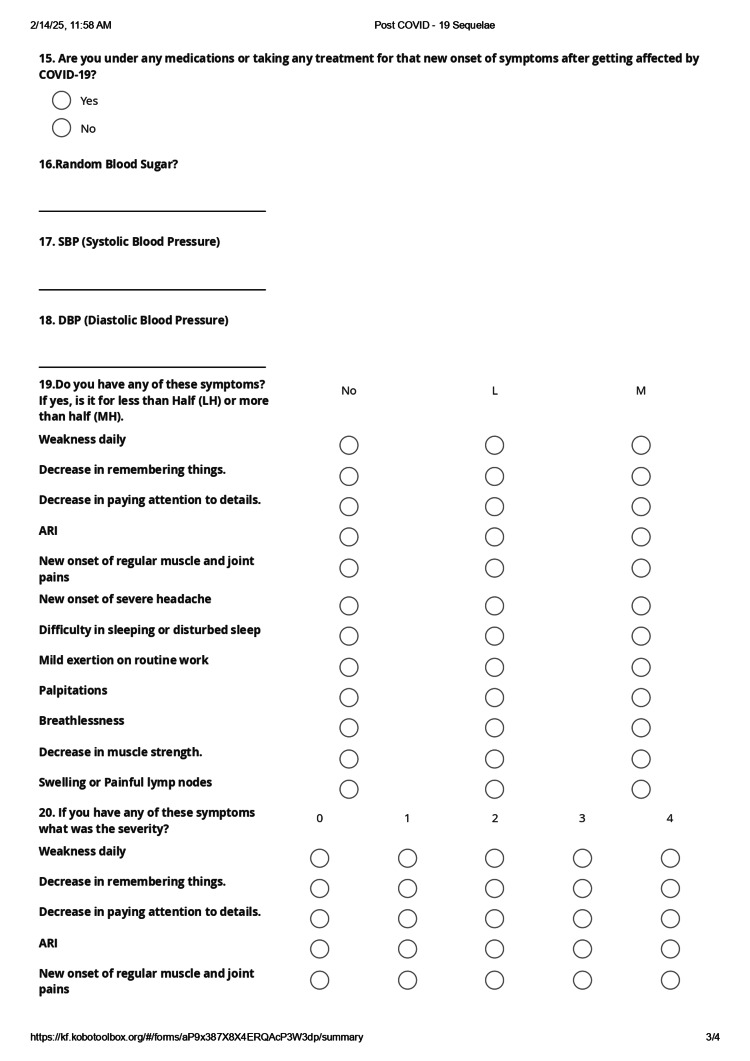
Post-COVID-19 questionnaire (page 3)

**Figure 6:**
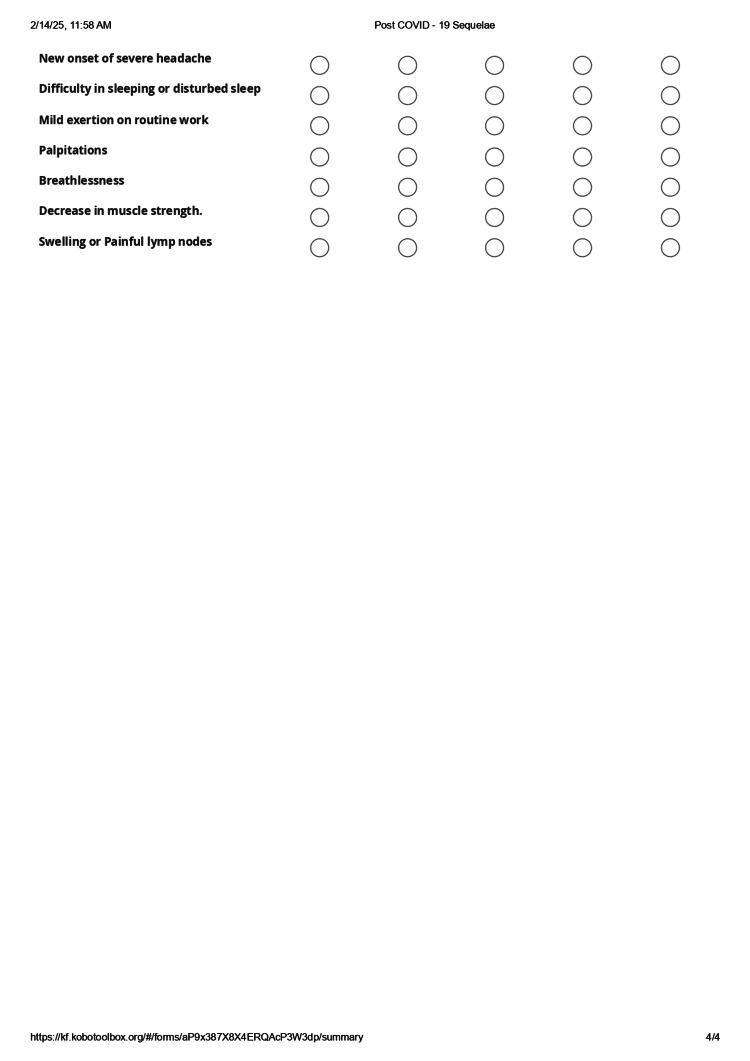
Post-COVID-19 questionnaire (page 4)
